# Domain and Switching Control of the Bulk Photovoltaic Effect in Epitaxial BiFeO_3_ Thin Films

**DOI:** 10.1038/s41598-019-50185-1

**Published:** 2019-09-27

**Authors:** David S. Knoche, Yeseul Yun, Niranjan Ramakrishnegowda, Lutz Mühlenbein, Xinye Li, Akash Bhatnagar

**Affiliations:** Zentrum für Innovationskompetenz SiLi-nano, Martin-Luther-Universität Halle-Wittenberg, Halle (Saale), 06120 Germany

**Keywords:** Nanoscience and technology, Ferroelectrics and multiferroics

## Abstract

Absence of inversion symmetry is the underlying origin of ferroelectricity, piezoelectricity, and the bulk photovoltaic (BPV) effect, as a result of which they are inextricably linked. However, till now, only the piezoelectric effects (inverse) have been commonly utilized for probing ferroelectric characteristics such as domain arrangements and resultant polarization orientation. The bulk photovoltaic effect, despite sharing same relation with the symmetry as piezoelectricity, has been mostly perceived as an outcome of ferroelectricity and not as a possible analytical method. In this work, we investigate the development of BPV characteristics, i.e. amplitude and angular dependency of short-circuit current, as the ferroelastic domain arrangement is varied by applying electric fields in planar devices of BiFeO_3_ films. A rather sensitive co-dependency was observed from measurements on sample with ordered and disordered domain arrangements. Analysis of the photovoltaic response manifested in a mathematical model to estimate the proportion of switched and un-switched regions. The results unravel the potential utility of BPV effect to trace the orientation of the polarization vectors (direction and amplitude) in areas much larger than that can be accommodated in probe-based techniques.

## Introduction

The observation of the photovoltaic (PV) effect in BiFeO_3_ (BFO) has revitalized interests in the field of photo-ferroics, i.e. in the interplay between ferroic orders and photo-electronic characteristics. The possibility to tune one by the other has laid the platform to conceptualize novel opto-electronic devices. Several investigations have considered the role of depolarization field and band bending at the electrode-film interface in separating the charge carriers for the resultant PV effect^1–4^. As a consequence, the PV effect in BFO has been demonstrated to be affected by the state of ferroelectric polarization^4–6^. This aspect has been conversely utilized to read the change in the state of polarization, i.e. up to down or vice versa^7^. However, for certain applications, mere knowledge about the sign of polarization is not sufficient, and information about the orientation is equally desired. This is particularly true for planar devices wherein the electric fields are applied in-plane. For instance, functioning of BFO-based magneto-electric devices largely depends upon the canted magnetic moment that is associated with the in-plane projection of the polarization^8^. Stripe-patterned ferroelastic domains separated by 71° domain walls in BFO films, with a large in-plane polarization component, have been successfully utilized to align the magnetic easy axis of the ferromagnetic top layer^9,10^. Recent discovery pertaining to electric field control of sykrmions in bi-layered heterostructures will motivate further research in such devices^11^. In all of these scenarios, knowledge about the in-plane polarization in terms of magnitude and orientation is necessary. Scanning probe techniques are typically used for this purpose, albeit with measurable areas limited to few tens of micrometers. Lately, second harmonic generation (SHG) has been also explored to detect the orientation of the in-plane polarization component^12^. Here, the absence of inversion symmetry in ferroelectric materials causes the impinging light waves to undergo frequency doubling. The emitted light wave can then be resolved to extract information about the polarization state, and distinguish between ordered and disordered domain configurations^12,13^.

Interestingly, another consequence of the absence of inversion symmetry is the generation of shift photocurrents under appropriate illumination^14^. The resultant photovoltaic effect is referred to as bulk photovoltaic (BPV) effect, and exhibits anomalous characteristics such as open-circuit voltages (*V*_*oc*_) that can well exceed the band gap of the material^15^. The photoresponse can be represented in the form of a tensor, which is analogous to the piezoelectric tensor and derived from the crystal symmetry. One of the critical consequences is the dependency of the PV current on the orientation of the linearly polarized light with respect to the measurement gap, and on BPV tensor coefficients *β*_ij_. In the case of single domain samples, and measurement along the polar axis, sinusoidal and cosinusoidal dependencies have been observed with a single tensor coefficient (*β*_22_) acting as a proportionality constant^16^. However, in the case of samples with poly-domain configurations, the resultant response can have contributions from multiple tensor coefficients. Mathematically it has been shown that the arrangement of polarization vectors of different domains results in such non-trivial solutions^17^. Therefore, it can be perceived that any change in the domain arrangement and net polarization will directly impact the BPV response. Conversely, the BPV effect can be also used to determine the domain arrangement and the effective state of polarization.

Till now, the BPV effect has been mostly studied in relation to single domain states with emphasis only on the observation rather than manipulation. In this work, we analyze the evolution of the BPV effect as the domains are switched upon the application of electric fields. The defining characteristics of the short-circuit current (*I*_*sc*_), viz. magnitude and angular dependence, were found to be surprisingly sensitive to the rotation of the in-plane net polarization (*P*_*net*_). Our analysis henceforth unravels the potential utility of the BPV effect in determining the orientation of *P*_*net*_ in areas of size that are not typically measurable with probe-based methods. We use planar top electrodes separated by a gap, which allows to observe the domain configuration using piezoresponse force microscopy (PFM) and to measure the corresponding BPV characteristics. Experiments were conducted with disordered and ordered domain arrangements.

## Results

**Figure 1:**
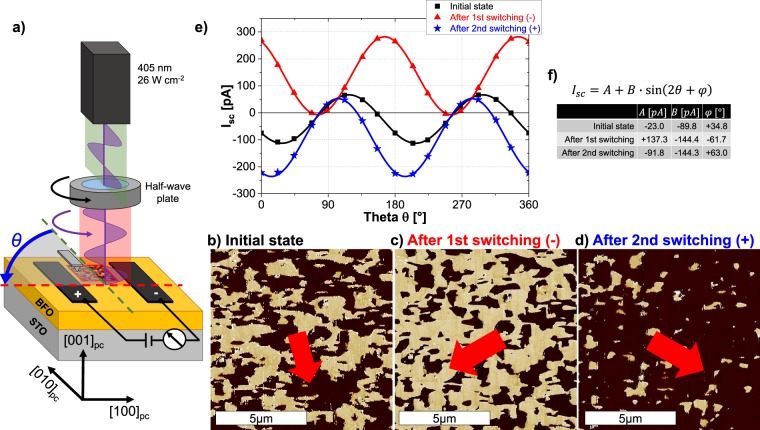
(**a**) Schematic setup of the measurements on BFO/STO: Orientation of linearly polarized laser light (green) is rotated by a half-wave plate by the angle *θ* (red). The beam spot is placed in between the electrodes, which are running along [010]_pc_. LPFM phase image (PFM probe along [010]_pc_, 10 × 10 µm^2^) of (**b**) the initial state, (**c**) after applying negative and (**d**) after applying positive electric fields, respectively. Red arrows indicate the direction of *P*_*net*_. (**e**) *I*_*sc*_ as a function of *θ* for the state shown in (**b**–**d**). Solid lines depict the fit of the data points with Eq. (1). (**f**) Values of *A*, *B* and $\phi$ extracted from fitting.

BFO films with a thickness of ~220 nm were deposited on (001) oriented SrTiO_3_ (STO) and (110) oriented DyScO_3_ (DSO) substrates using pulsed laser deposition. The respective samples will be referred to as BFO/STO and BFO/DSO. The resultant topography of the samples and the corresponding X-ray diffraction 2*θ*/$\omega$-scans prove the epitaxial relation between the film and substrate. The details are provided in Supplementary Fig. S1. Planar electrodes, with a length of 950 µm and separated by approximately 40 µm, were fabricated on the surface of the samples. The setup of the photoelectrical (PE) and PFM measurements is shown schematically in Fig. 1a. PE measurements were performed at room temperature with a diode laser of wavelength 405 nm (3.05 eV) as the illumination. The orientation of the linearly polarized light was rotated using a half-wave plate. The angle *θ* represents the orientation of electric field plane of the light with respect to the [010]_pc_ direction. The short-circuit current *I*_*sc*_ can be represented with the following equations^17^:

1 $$\begin{array}{llll}{{\bf{P}}}_{{\bf{n}}{\bf{e}}{\bf{t}}}\parallel {\bf{e}}{\bf{l}}{\bf{e}}{\bf{c}}{\bf{t}}{\bf{r}}{\bf{o}}{\bf{d}}{\bf{e}}{\bf{s}}\,: & {I}_{sc}(\theta ) & = & E{A}_{cs}(\tfrac{{\beta }_{33}}{3\sqrt{3}}-\tfrac{{\beta }_{31}}{3\sqrt{3}}+\tfrac{2{\beta }_{22}}{3\sqrt{6}}+\tfrac{{\beta }_{15}}{3\sqrt{3}})\,\sin (2\theta +\phi )\\  &  & = & A+B\,\sin (2\theta +\phi )\end{array}$$

2 $$\begin{array}{llll}{{\bf{P}}}_{{\bf{n}}{\bf{e}}{\bf{t}}}\perp {\bf{e}}{\bf{l}}{\bf{e}}{\bf{c}}{\bf{t}}{\bf{r}}{\bf{o}}{\bf{d}}{\bf{e}}{\bf{s}}\,: & {I}_{sc}(\theta ) & = & E{A}_{cs}(\frac{{\beta }_{33}}{3\sqrt{3}}+\frac{2{\beta }_{31}}{3\sqrt{3}}-\frac{{\beta }_{22}}{3\sqrt{6}}+\frac{{\beta }_{15}}{3\sqrt{3}})\\  &  &  & +\,E{A}_{cs}(\frac{2{\beta }_{22}}{\sqrt{6}}+\frac{{\beta }_{15}}{\sqrt{3}})\,\sin (2\theta +\phi )\\  &  & = & C+D\,\sin (2\theta +\frac{\pi }{2}+\phi )\\  &  & = & C+D\,\cos (2\theta +\chi )\end{array}$$

The equations have been derived for BFO films with an ordered array of domains that are separated by 71° domain walls. To simplify the equations, we have replaced the associated constants, namely, BPV coefficients *β*_ij_, light intensity *E* and the cross-sectional area *A*_*cs*_, with condensed notations *B*, *C* and *D*. Angles $\phi$ and $\chi$ have been added in Eqs (1) and (2), respectively, to incorporate any tilt of *P*_*net*_ with respect to the electrodes. In addition, *A* has been included as an offset in Eq. (1), and bears no physical relevance.

Due to the in-plane compressive strain and homogeneous TiO_2_ termination of the STO substrate, only four out of the eight possible domain variants are present that are uniformly aligned along the out-of-plane direction^18–20^ and the out-of-plane component of the polarization does not change throughout our experiments. Therefore, only the lateral PFM (LPFM) phase images will be henceforth presented. Figure 1b shows the disordered domain arrangement in the initial state of the BFO/STO sample wherein the ratio of dark to bright regions is ~55:45. An additional LPFM phase image was acquired with the cantilever aligned along the [100]_pc_ direction and is shown in Supplementary Fig. S2. The dark-to-bright ratio is around ~65:35. It can be inferred from these images that *P*_*net*_ is tilted within the electrode gap, as indicated by the red arrow in Fig. 1b. The subsequent PE measurement (Fig. 1e, black) resulted in an *I*_*sc*_ and *V*_*oc*_ that varies as the electric field plane of the light is rotated by an angle *θ* (*V*_*oc*_ values of the investigated samples can be found in Supplementary Fig. S3). This proves the dominance of the BPV effect in these samples. The variations in *I*_*sc*_ are in good agreement with Eq. (1) which describes the PV current when *P*_*net*_ is parallel to the electrodes. The measured values of the current were analyzed with Eq. (1) and the respective coefficients extracted from the fitting are summarized in Fig. 1f. The values of $\phi$, *A* and *B* are 35°, 23 pA and 89.9 pA, respectively. The non-zero value of coefficient *A* and the substantial value of $\phi$ suggest a tilt of *P*_*net*_ with respect to the electrodes, as was also inferred from the PFM analysis. In the next step, electric fields were applied across the electrodes to rearrange the domain configuration and associated *P*_*net*_. Negative fields were applied and PFM scans were acquired from the same area as in Fig. 1b. The LPFM phase image (Fig. 1c) clearly depicts an evident reduction of the dark-to-bright ratio which confirms the tendency of *P*_*net*_ to align with the direction of the electric field. The subsequent PE measurement reveals a shift of *I*_*sc*_(*θ*) along the *y*- and *x*-axis (Fig. 1e, red). The coefficients *A* and $\phi$ undergo a change in their respective signs, while *B* attains a higher value. An increment in *B* is related to an overall higher magnitude of *P*_*net*_ which is manifested by a higher ordering of the domains after the switching process. On the other hand, $\phi$ approaches 90° in conjunction with a higher value of *A*. Therefore, the PV response seems more alike the relation given in Eq. (2), which is valid when *P*_*net*_ is perpendicular to the electrodes. Thereafter, positive electric fields were applied and the rearranged domain configuration from the same area is shown in Fig. 1d. Apparently, the dark-to-bright ratio exceeds the value of the initial state. The analysis of the PV response with Eq. (1) results in a similar value of *B* suggesting a nearly unchanged magnitude of *P*_*net*_. Coefficients $\phi$ and *A* undergo a change of sign which largely emulates a flip of *P*_*net*_ to align along the direction of the electric field, although the magnitudes slightly differ. A possible reason for the difference could be a non-uniform switching arising from inhomogeneous electric fields close to the edges of the surficial top electrodes^21^ and the large size of the gap. From these observations it can be proposed that the analysis of the BPV response with Eqs (1) and (2) present an efficient framework to determine the orientation of the polarization. The extracted coefficients contain information about the orientation and magnitude of *P*_*net*_. Also, noteworthy to mention is the size of the gap under consideration, 40 × 950 µm^2^, which is much larger than the areas typically measurable in a single image scan of a probe-based technique such as PFM. However, to gain further insight into the intricate correlations, it is imperative to study the PV response from samples which have a long-range ordering of domains with a known direction of *P*_*net*_.

**Figure 2:**
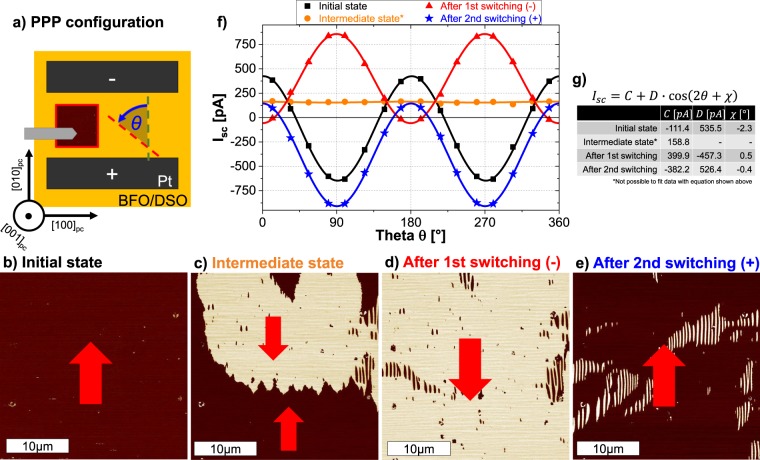
(**a**) Schematic setup of the measurements in the PPP configuration (*P*_*net*_ perpendicular to electrodes) on BFO/DSO. LPFM phase image (PFM probe along [100]_pc_, 30 × 30 µm^2^) (**b**) of the initial state, (**c**) of intermediate state (negative electric fields applied), (**d**) after applying higher negative and (**e**) after applying positive electric fields, respectively. Red arrows indicate the direction of *P*_*net*_. (**f**) *I*_*sc*_ as a function of *θ* for the state shown in (**b**–**e**). Solid lines depict the fit of the data points with Eq. (2). (**g**) Values of *C*, *D* and $\chi$ extracted from fitting.

For this purpose, BFO films grown on DSO substrates, exhibiting 71° domain walls and *P*_*net*_ pointing along the [010]_pc_-direction, were used. Prior investigations were performed to determine the direction of the polarization, and electrodes were aligned perpendicular (PPP configuration) and parallel (PPL configuration) to *P*_*net*_. Figure 2a schematically depicts the PE and PFM measurement in the PPP configuration. Figure 2b is a representative LPFM image acquired from in between the gap with the PFM probe aligned along [100]_pc_. The uniform color confirms the direction of *P*_*net*_ perpendicular to the electrodes. The typical stripe-like pattern associated with the 71° domain walls was also imaged but with the probe aligned along [010]_pc_, and will be discussed later. The PE measurement from the initial state demonstrates a rather excellent agreement between the *I*_*sc*_ and Eq. (2) (Fig. 2f,g), with the angular offset $\chi$ bearing a minimal value of 2°.

Thereafter, the gap was switched to an intermediate state to have a nearly equal proportion of areas with oppositely aligned *P*_*net*_, as shown in Fig. 2c. Additional LPFM images revealing the presence of a global domain wall between switched and unswitched regions are shown in Supplementary Fig. S4. The resultant PV response loses the cosinusoidal *θ*-dependency and a steady *I*_*sc*_ of around 160 pA was measured. The comparison with Eq. (2) suggests an apparent drop in the value of *D*, which comprises of *β*_22_ and *β*_15_. The measured *I*_*sc*_ can then be attributed primarily to the *θ*-independent quantity *C* which has additional contributions from *β*_31_ and *β*_33_. Subsequently, higher electric fields were applied with the objective to completely switch the gap, and the LPFM phase image in Fig. 2d confirms this scenario. The corresponding PV response regains the cosinusoidal dependency on *θ* with coefficients *C* and *D* bearing high values, and $\chi$ assuming a value of 180° implying a complete inversion of *P*_*net*_. The variation in the values of *D* (i.e. 535 pA in the initial state → zero in the intermediate state → −457 pA after first switching) clearly mimics the magnitude of *P*_*net*_. To further confirm this, electric fields were applied to switch the gap back to the initial state (Fig. 2e). As expected, a nearly identical value of *D* was obtained. The relevance of *C* is also worthy to be mentioned. The sign of *C* clearly follows the direction *P*_*net*_, but the value seems to have a correlation with the domain width (size of domain in the [010]_pc_-direction) which was observed to be significantly larger as new domains were formed (see Supplementary Fig. S5). Thus it can be concluded that the switching process in the PPP configuration does not involve any substantial rotation of *P*_*net*_. The antiparallel and parallel alignment of the electric field with respect to *P*_*net*_ only leads to a lowering and subsequent gain of the magnitude of *P*_*net*_.

**Figure 3:**
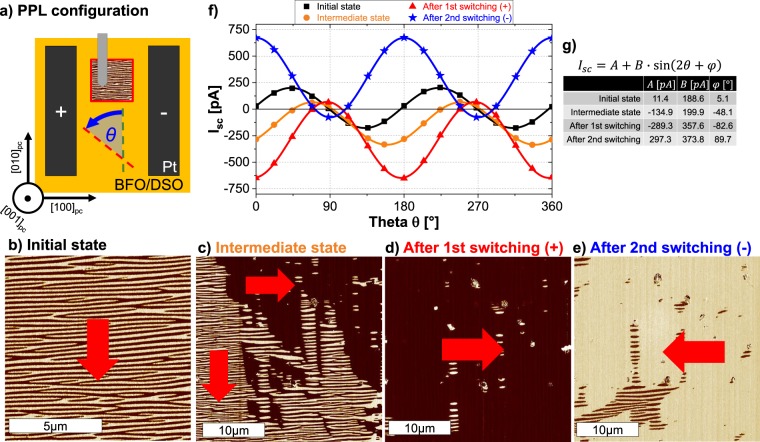
(**a**) Schematic setup of the measurements in the PPL configuration (*P*_*net*_ parallel to electrodes) on BFO/DSO. LPFM phase image (PFM probe along [100]_pc_) (**b**) of the initial state, (**c**) of intermediate state (negative electric fields applied), (**d**) after applying higher negative and (**e**) after applying positive electric fields, respectively. Red arrows indicate the direction of *P*_*net*_. (**f**) *I*_*sc*_ as a function of *θ* for the state shown in (**b**–**e**). Solid lines depict the fit of the data points with Eq. (1). (**g**) Values of *A*, *B* and $\phi$ extracted from fitting.

**Figure 4:**
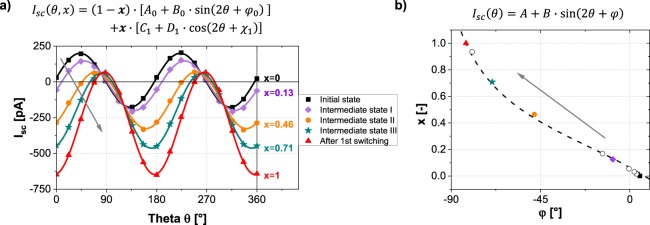
(**a**) *I*_*sc*_ as a function of *θ* in the initial state (black), in the intermediate state (purple, orange, green) and after applying the highest (positive) electric field (red). Data points correspond to the PE measurements at certain angles *θ* fitted with Eq. (3) (solid lines). Parameter × represents the extent of switching. (**b**) The extent of switching *x* plotted as a function of the angle $\phi$ (acquired from fitting measured values with Eq. (1)) for the initial state, several intermediate states, and the state after the first switching. The fit emphasizes the possibility to use $\phi$ as a probing parameter to measure the extent of switching and thus the tilt of *P*_*net*_ (Derivation of fitting function can be found in the Supplementary Information).

For the next set of experiments, the PPL configuration (Fig. 3a) was used, which apparently also allowed us to observe and investigate the rotation of *P*_*net*_. The initial state of the gap was confirmed to have *P*_*net*_ aligned parallel to the electrodes with the imaging of long stripe like patterned domains along [100]_pc_ (Fig. 3b). As a result, the PV response was found to be in good agreement with Eq. (1) with minimal values of coefficient *A* and angle $\phi$ of around 11 pA and 5°, respectively. Upon the application of a moderate electric field, the *P*_*net*_ in some regions aligns along the electric field, while in other regions remains unchanged (Fig. 3c). Therefore, the gap adopts an intermediate state with *P*_*net*_ tilted by an angle of ~45°. Precisely this scenario is also visible in the PV response wherein the angle $\phi$ assumes a value of 48.1°. As higher fields are applied, the gap uniformly switches (Fig. 3d) and the PV response changes from sinusoidal to cosinusoidal shape. This change is related to an orthogonal rotation of *P*_*net*_ and is illustrated by the change of $\phi$ to 82.6° and *A* assuming a non-negligible value of −289 pA. Applying electric fields of opposite polarity confirms this (Fig. 3e), and likewise the PPP case, a PV response with similar values of *A* and *B* is observed with $\phi =89.7^\circ$. It is evident from these measurements, that the electric field forces *P*_*net*_ to re-orient stepwise from the parallel to a perpendicular configuration. This situation can be effectively modeled by combining Eqs (1) and (2). The resultant equation is of the type:

3 $${I}_{sc}(\theta ,x)=(1-x)\cdot [{A}_{0}+{B}_{0}\,\sin (2\theta +{\phi }_{0})]+x\cdot [{C}_{1}+{D}_{1}\,\cos (2\theta +{\chi }_{1})]$$

wherein *x* and (1 − *x*) represent the fraction of area with changed and unchanged orientation of *P*_*net*_, respectively. To investigate the validity of this relation, first, a series of PE measurements were conducted to acquire PV responses from differently switched states of the gap. The values of the coefficients *A*_*0*_, *B*_*0*_, ${\phi }_{0}$ and *C*_*1*_, *D*_*1*_, ${\chi }_{1}$ were determined from the initial (PPL) and final (PPP) state, respectively. Thereafter, all the responses were analyzed with Eq. (3), by keeping the coefficients fixed and allowing the variation of only *x*. All of the acquired PV responses are in excellent agreement with Eq. (3) as visible in Fig. 4a, which also allows to extract information about the proportion of the switched area *x*. In addition, the intermediate switched states were analyzed with Eq. (1) to acquire the different values of angle $\phi$. The plot between *x* and $\phi$ is shown in Fig. 4b and demonstrates the sensitivity of the BPV response to smallest changes in the orientation of *P*_*net*_. Hence we can propose that the angle $\phi$ can be effectively utilized as a probing parameter for *P*_*net*_. Assuming an ideal alignment of *P*_*net*_ in the initial state, the angle $\phi$ can vary from 0° to ±90° irrespective of the PV current magnitudes. The sign associated with $\phi$ explicitly is related to the direction of *P*_*net*_ in reference to the electrodes, while the absolute value indicates the extent of the switching and the connected tilt angle of *P*_*net*_. This is also analogous to the phase of the locked-in signal measured in PFM which undergoes a ~180° phase shift as domains with opposite polarization are scanned. An analogous description of the switching progress in the PPP configuration is shown in Supplementary Fig. S6.

## Discussion

With the above results in perspective, we are now in a position to draw some comparisons with the present state of knowledge and discuss some applicability issues. Earlier studies have demonstrated a switchable PV effect in BFO films in capacitor or out-of-plane geometries^6,22^. However, the origin of the PV effect in such scenarios has been found to be susceptible to the ferroelectric-electrode interface, and also to the overall conduction of the gap^1^. As a result, *I*_*sc*_ can be merely utilized to determine the direction of polarization.

The planar geometries used in this work allowed us to focus on the *I*_*sc*_ originating from the BPV effect, which is evident from the angular-dependent characteristics. Similar planar geometries have been used in the past for studying photo-related processes in BFO films^15,23–28^. However, the use of either white light or light with fixed plane of polarization restricted the studies to switching of ferroelectricity and observation of switchable PV effect^23–26^. Likewise the vertical or capacitor geometries, even in planar configuration high electric fields can result in other effects as well, such as migration of oxygen vacancies and accumulation of charges at the electrode-ferroelectric interface^24^. In addition, domain walls separating head-to-head domains can be created which haven been demonstrated to a show sub-band gap photoresponse^25,26^. But large sized gaps and above band-gap illumination (necessary for BPV effect) allowed us to circumvent such secondary contributions and focus only on the bulk photoresponse. This is also evident from *I*_*sc*_ and *V*_*oc*_ measurements which always exhibited an angular dependency on light polarization, i.e. in pristine, semi-and completely switched states.

Our analysis of *I*_*sc*_, in conjunction with the domain arrangement, provided an insight into the evolution of the BPV effect. The responses were compared with previously calculated relations, which manifested the extraction of crucial coefficients. Henceforth, a model was proposed which can be utilized for estimating not only the orientation and magnitude of *P*_*net*_, but also the proportion of switched regions. The measured areas are much larger than the areas that can be typically accommodated in a single image scan of a probe-based technique. These results therefore unravel the potential of the BPV phenomenon, typically perceived as an outcome of domain and polarization configuration, as a means of detection. The simplicity of the method presented in this work can be much appreciated upon comparison with the well-established SHG technique. In addition, the possibility to estimate the proportion of switched/un-switched region is yet to be demonstrated with SHG based methods.

However, one of the main drawbacks of this method is the necessity of the ferroelectric to exhibit BPV characteristics. But, as per rules of symmetry, all ferroelectric materials are capable of such an effect. For instance, anomalous photoresponse has been reported and studied in LiNbO_3_^29^, KNbO_3_^16^, BaTiO_3_^30^ and PbTiO_3_^31^. Therefore, further calibrations will certainly strengthen the framework presented here for the determination of polarization related parameters in a variety of ferroelectric materials.

## Methods

### Thin film growth

The BiFeO_3_ thin films were grown on single crystalline SrTiO_3_ and DyScO_3_ substrates using a pulsed laser deposition system (SURFACE PLD-Workstation). During deposition, the substrate was kept at 625 °C and exposed to an oxygen partial pressure of 0.145 mbar. The KrF excimer laser was set to an energy density of ~1.45 J cm^−2^ with a pulse frequency of 2 Hz.

### Electrode deposition

The electrodes were structured using a standard photolithography process. DC sputtering was used to deposit the electrode material (Pt:Pd, 80:20) with a thickness of ~70 nm.

### Piezoresponse force microscopy

PFM images were acquired with a Park NX10 system combined with an external lock in amplifier (Zurich Instruments). The AC voltage (3 V, 20 kHz) was applied through a cantilever equipped with a platinum coated tip (MikroMasch NSC 14).

### Photoelectrical measurements

A high impedance electrometer (Keithley 6517B) acted as a voltage source (IV-characteristics, switching voltage) and simultaneously measured the current. The samples were illuminated by a diode laser (Cobolt 06 MLD) with a wavelength of 405 nm and 100 mW power.

### Switching experiments

During the switching process the maximum applied voltage was increased in 50 V steps. The voltage was ramped up from zero to the maximum voltage of the switching step with a rate of 5 V s^−1^. IV characteristics were acquired after each switching step for a fixed angle *θ*. PFM measurement and *θ*-dependent PE measurements were performed after certain switching steps.

## Supplementary information

Supplementary Material: Domain and Switching Control of the Bulk Photovoltaic Effect in Epitaxial BiFeO3 Thin Films
