# Supplementary material for: Domain and Switching Control of the Bulk Photovoltaic Effect in Epitaxial BiFeO3 Thin Films
Source: Sci Rep. 2019 Sep 27;9:13979. doi: 10.1038/s41598-019-50185-1 (PMC6765050; doi:10.1038/s41598-019-50185-1)
Supplement: Supplementary file 1 — Supplementary Material: Domain and Switching Control of the Bulk Photovoltaic Effect in Epitaxial BiFeO3 Thin Films [file 41598_2019_50185_MOESM1_ESM.pdf]

Supplemental Material: Domain and Switching Control of the Bulk Photovoltaic Effect in Epitaxial $BiFeO_3$ Thin Films

David S. Knoche, Yeseul Yun, Niranjan Ramakrishnegowda, Lutz Mühlenbein, Xinye Li, and Akash Bhatnagar*

Zentrum für Innovationskompetenz SiLi-nano,
Martin-Luther-Universität Halle-Wittenberg,
Karl-Freiherr-von-Fritsch-Str. 3, 06120 Halle (Saale), Germany
*akash.bhatnagar@physik.uni-halle.de

May 20, 2019

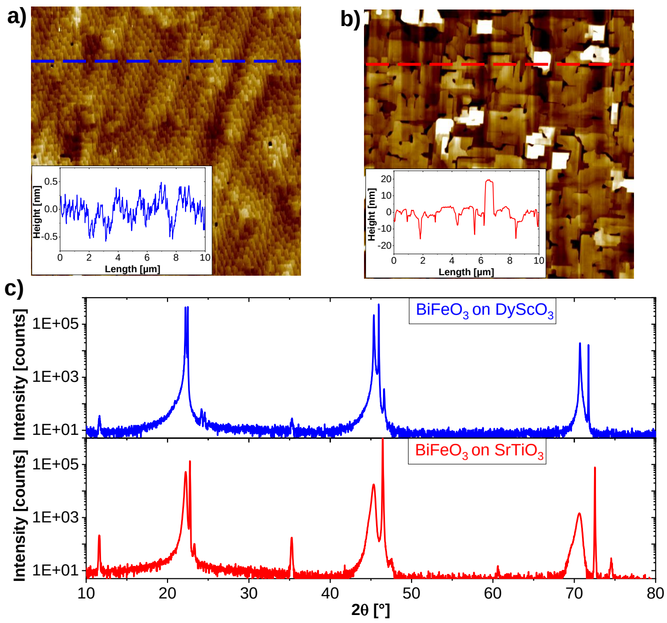

Figure S1: Topography scan (10 x 10 µm^2) of (a) BFO/DSO and (b) BFO/STO sample acquired by AFM. The insets show the respective line profile along the dotted line. (c) X-ray diffraction $2\theta/\omega$-scan of both samples.

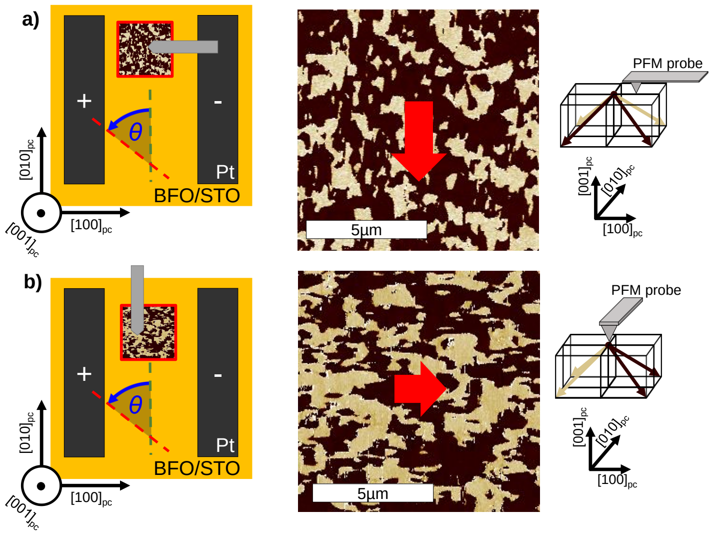

Figure S2: LPFM phase images of the BFO/STO when PFM probe was aligned along (a) $[100]_{\mathrm{pc}}$ and (b) $[010]_{\mathrm{pc}}$, respectively, reveal different dark-to-bright ratios.

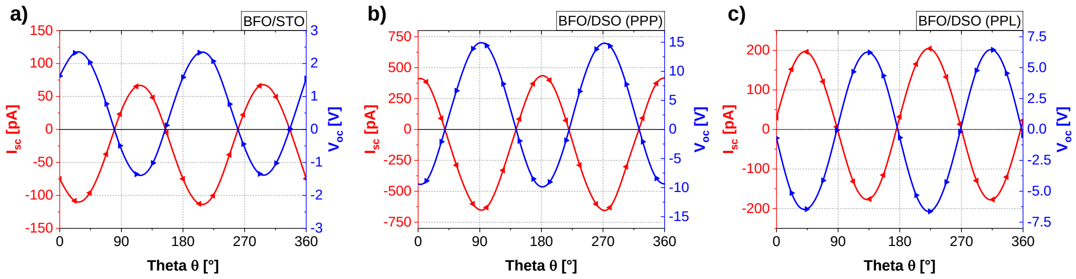

Figure S3: I_{sc} and V_{oc} as a function of θ in the initial state of (a) BFO/STO, (b) BFO/DSO PPP configuration and (c) BFO/DSO PPL configuration.

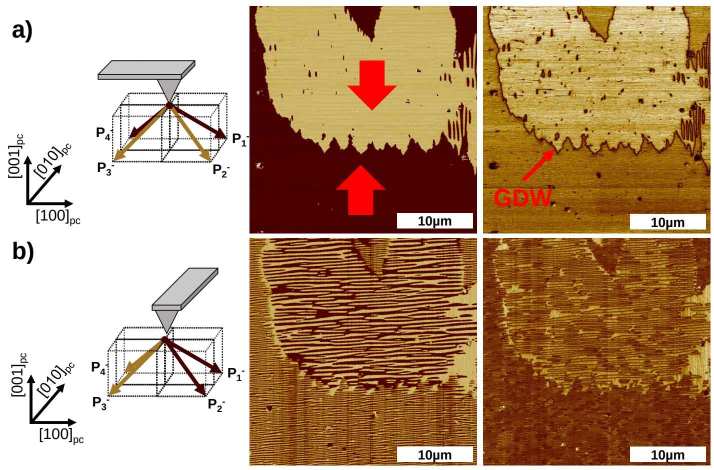

Figure S4: LPFM phase and amplitude images of the intermediate switching state, when cantilever was aligned along a)$[100]_{\mathrm{pc}}$ and b)$[010]_{\mathrm{pc}}$, respectively. The global domain wall (GDW) separates switched and unswitched regions with opposite direction of P_{net}.

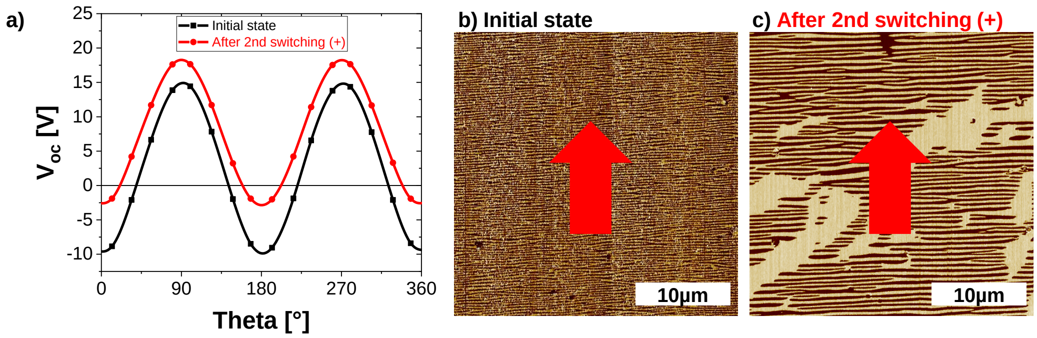

Figure S5: a) V_{oc} as function of θ of the initial state and after the second switching. The maximum value increased from ∼15 V to ∼18 V. The corresponding LPFM phase images show an increased domain width (c) after the 2nd switching compared to (b) the intial state.

Additional Information FIG. 4 - Derivation of Fitting Function

$$\textbf{Equation 1} = \textbf{Equation 3 (Main text)}$$

$$\Leftrightarrow A + B\sin(2\theta+\varphi) = (1-x)(A_0 + B_0\sin(2\theta+\varphi_0)) + x(C_1 + D_1\cos 2\theta + \chi_1)$$

$$\Rightarrow x(\varphi) = \left[1 + \frac{D_1(\sin(\chi_1) + cos(\chi_1)\tan\left(\frac{\pi}{2}-\varphi\right))}{B_0(\cos(\varphi_0) - \sin(\varphi_0)\tan\left(\frac{\pi}{2}-\varphi\right))}\right]^{-1} \tag{S1}$$

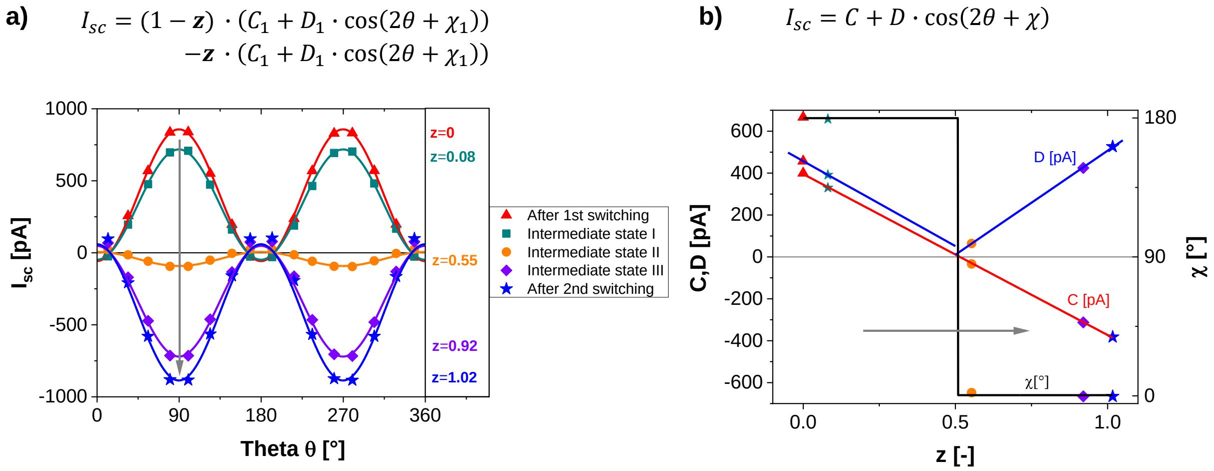

Figure S6: (a) I_{sc} as a function of θ for switching steps of within the second switching cycle (positive electric fields). Parameters z indicates the extent of switching extracted from fitting with the equation shown above the graph. (b) Corresponding values of C, D and χ extracted from fitting with Equation 2 as a function of the extent of switching z.
